# Effects of parietal iTBS on resting-state effective connectivity within the frontoparietal network in patients with schizophrenia: An fMRI study

**DOI:** 10.1016/j.nicl.2024.103715

**Published:** 2024-11-26

**Authors:** Li Li, Lina Wang, Han Wu, Bing Li, Weigang Pan, Wenqing Jin, Wen Wang, Yanping Ren, Chaomeng Liu, Xin Ma

**Affiliations:** a Beijing Key Laboratory of Mental Disorders, National Clinical Research Center for Mental Disorders & National Center for Mental Disorders, Beijing Anding Hospital, Capital Medical University, Beijing, PR China; b Advanced Innovation Center for Human Brain Protection, Capital Medical University, Beijing, PR China; c Hebei Provincial Mental Health Center, Baoding, PR China; d Hebei Key Laboratory of Major Mental and Behavioral Disorders, Baoding, PR China; e The Sixth Clinical Medical College of Hebei University, Baoding, PR China

## Abstract

**Background:**

Although intermittent theta burst stimulation (iTBS) has shown effectiveness in addressing working memory (WM) deficits in individuals with schizophrenia (SZ), the current body of evidence is limited and the specific mechanisms involved remain unclear. Therefore, this pilot fMRI study aimed to examine the efficacy of parietal iTBS in ameliorating WM impairments and explore its influence on the resting-state effective connectivity within the frontoparietal network in patients with SZ.

**Method:**

A total of 48 patients diagnosed with SZ were randomly assigned to an active or sham iTBS group and underwent 20 sessions of active or sham iTBS over 4 weeks. Subsequently, all patients underwent cognitive tests, clinical symptom assessments, and resting-state functional MRI (rs-fMRI) scans. The effective connectivity between the frontal and parietal brain regions during the rs-fMRI scans was analyzed using a spectral dynamic causal modeling approach. Additionally, this trial was registered at the Chinese Clinical Trial Registry in November 2022 (registry number: ChiCTR2200057286).

**Results:**

iTBS treatment improved the positive symptoms, negative symptoms, general psychopathology, and WM deficits. Following the iTBS intervention, the active group demonstrated a significant increase in connectivity strengths from the right MFG to the right SPL (*p* = 0.031) and from the left SPL to the left MFG (*p* = 0.010) compared to the pre-treatment levels. Additionally, compared to the sham group, the active group displayed a significantly higher connectivity strength from the right MFG to the right SPL (*p* = 0.042) after iTBS treatment.

**Conclusion:**

All these findings suggest that iTBS targeting the parietal region may influence the resting-state effective connectivity within the frontoparietal network, thereby offering promising therapeutic implications for alleviating the cognitive deficits in SZ.

## Highlights

• First use of parietal iTBS to treat working memory deficits in patients with schizophrenia.
• Parietal iTBS treatment alters the resting-state connectivity of the frontoparietal network.
• iTBS targeting the parietal region shows promise for treating cognitive impairment in schizophrenia.

## 1 Introduction

Schizophrenia (SZ) is a severe psychiatric disorder affecting approximately 21 million individuals globally. This complex mental disorder presents with varied symptoms, such as delusions, hallucinations, disorganized speech, abnormal psychomotor behavior, and cognitive impairment (Charlson et al., 2018, Heckers et al., 2013). Although antipsychotic medications have proven efficacious in managing positive symptoms, their effectiveness in ameliorating cognitive dysfunction is limited (Elkis and Buckley, 2016, Keefe et al., 2007). Cognitive deficits manifest in the prodromal phase of SZ and persist throughout the disease course, with their presence observed even after the resolution of clinical symptoms (Arsenault-Mehta et al., 2024, Javitt, 2023). Working memory (WM) impairment is one of the core cognitive deficits, with noteworthy implications for restoring social functioning in individuals with SZ (Gold and Luck, 2023). Thus, prioritizing the improvement of WM deficits should be a crucial objective in treatment interventions designed to improve functional outcomes in this patient population.

WM is characterized as a cognitive system involved in the temporary storage and manipulation of information, encompassing three distinct phases: encoding, retention, and information recall (Baddeley, 1992, Baddeley et al., 2001). Meta-analyses of neuroimaging studies have consistently demonstrated frontoparietal activation during WM tasks across various paradigms and modalities, highlighting the core role of this region in the WM network (Nielsen et al., 2017, Rottschy et al., 2012). For instance, a prior investigation revealed that individuals experiencing first-episode psychosis had reduced activation in the middle frontal gyrus (MFG) and superior parietal lobule (SPL) during n-back WM tasks compared to the activation levels in those without psychiatric disorders, indicating differences in the underlying brain connectivity (Dauvermann et al., 2013). Moreover, a previous study observed that the WM-related connectivity between the right MFG and SPL progressively decreased from healthy controls to individuals at ultra-high risk for psychosis and ultimately to untreated patients experiencing their first episode of psychosis (Schmidt et al., 2013).

Current pharmacological interventions have been ineffective in alleviating WM impairment in the SZ population (Wang et al., 2024). In recent years, high-frequency repetitive transcranial magnetic stimulation (rTMS) has been increasingly utilized in the neurocognitive research field as a non-invasive technique for investigating brain function (Luber and Lisanby, 2014, Mikellides et al., 2021). Two earlier *meta*-analyses have assessed the efficacy of rTMS in treating WM deficits in patients with SZ (Hyde et al., 2022, Jiang et al., 2019). Among them, one *meta*-analysis reported that rTMS targeting the left dorsolateral prefrontal cortex (DLPFC) could significantly improve WM performance in patients with SZ (Jiang et al., 2019). In contrast, the second *meta*-analysis did not reveal any positive impact of rTMS on WM impairments (Hyde et al., 2022). A previous study administered rTMS on the left DLPFC or midline parietal regions and found that solely stimulating the parietal region significantly decreased the response time during WM tasks without affecting accuracy (Luber et al., 2007). Furthermore, several researchers have suggested that parietal cortex dysfunction is a critical factor in WM storage deficits in patients with SZ (Benson and Park, 2013, Haenschel et al., 2007, Hahn et al., 2018).

Excitatory intermittent theta burst stimulation (iTBS) is a novel rTMS technique that can induce long-term potentiation, a form of synaptic plasticity (Rossini and Rossi, 2007, Wu et al., 2020) and substantially reduces treatment duration and healthcare expenses, thus making it a promising option with numerous clinical uses (Liu et al., 2023a). Recent studies have revealed that iTBS can effectively improve clinical symptoms and cognitive deficits associated with SZ, underlining its potential therapeutic value (Chen et al., 2019, Wang et al., 2020). In this study, we conducted a randomized sham-controlled trial to investigate the impact of parietal iTBS on WM deficits in individuals with stable SZ. Functional connectivity analyses are limited to exhibiting the synchronization of time series among spatially separate regions, thus lacking information concerning the directionality of the connections. In contrast, effective connectivity analyses show the direction as well as the valence of the connections (Friston et al., 2014, Razi et al., 2015). Therefore, this study also examined the potential modulation effects of iTBS on the resting-state effective connectivity in the frontoparietal network using a spectral dynamic causal modeling (spDCM) approach. We hypothesized that iTBS would improve WM deficits in patients with stable SZ, potentially via the modulating influence of iTBS on the frontoparietal network.

## 2 Materials and Methods

### 2.1 Study patients

Patients aged 18–45 years were recruited and screened for study eligibility according to the following inclusion criteria: (1) education years ≥ 9 years; (2) right-hand dominant; previous research had indicated that, among individuals with schizophrenia, non-right-handedness was correlated with learning disabilities, and mixed-handedness was linked to the manifestation of positive symptoms (Mallet et al., 2022). These associations may be indicative of atypical neural developmental patterns (Geschwind and Galaburda, 1985). (3) met the diagnostic criteria for SZ according to the 5th edition of the Diagnostic and Statistical Manual of Mental Disorders (DSM-V); (4) received stable psychotropic medication for ≥ 8 weeks before the study, with no need for medication adjustment (Wang et al., 2022); (5) not receiving electroconvulsive therapy for approximately 6 months (Wang et al., 2022); and (5) provided informed consent. The patient exclusion criteria were as follows: (1) a history of intracranial cerebrovascular disease, brain damage, epilepsy, or neurodegenerative disease; (2) severe systemic illness or other major psychiatric conditions (e.g., intellectual disability, depressive disorder, or bipolar disorder) (Liu et al., 2022) (2) MRI contraindications; (3) previously participated in a similar TMS trial; or (4) the presence of an implant device contraindicated for MRI or TMS (e.g., deep brain stimulator or cardiac pacemaker).

This study was approved by the ethics committees of Hebei Provincial Mental Health Center and Beijing Anding Hospital, Capital Medical University. Additionally, this trial was registered at the Chinese Clinical Trial Registry in November 2022 (registry number: ChiCTR2200057286).

### 2.2 Study intervention

Forty-eight eligible patients were randomly allocated to an active iTBS or sham rTMS group at a 1:1 ratio. The sequence of allocation was generated using SPSS 26.0 software (SPSS, Chicago, IL, USA) by an independent staff member who was not involved in participant recruitment, intervention administration, outcome assessment, or statistical analysis. Furthermore, the outcome assessors and statistical analysts were blinded to the group allocations. The iTBS intervention was conducted using Super Rapid 2 stimulator (Magstim, Wales, UK) with a standard 70-mm figure-of-eight coil (Active Air film coil, Maximum 100 Hz, Magstim, Wales, UK). The employed iTBS protocols have been established to be safe and feasible for clinical practice (Liu et al., 2023a). The stimulation sites for iTBS in this study were located at P3 of the parietal lobe based on the 10–20 EEG system (Herwig et al., 2003). Please refer to Supplementary text 1 for specific operating steps. The muscle response of the abductor pollicis brevis in the right hand was utilized to ascertain the resting motor threshold (RMT), which was established at 80 %. In the active group, the iTBS protocol comprised three 50 Hz stimuli delivered at a rate of 5 Hz for a duration of 2 s on and 8 s off, with each session consisting of a total of 600 pulses over 190 s per day as recommended by Huang and colleagues (Huang et al., 2005). The sham group underwent the same iTBS intervention; however, the coil was inclined at a 90° inclined coil to minimize neural stimulation while maintaining auditory artifacts. All patients underwent 20 sessions of active or sham iTBS five times per week over 4 weeks.

### 2.3 Assessment of clinical symptoms and neuropsychological function

The clinical symptoms of the patients were assessed by trained psychiatrists using the Positive and Negative Symptom Scale for SZ (PANSS). The PANSS instrument has 30 items, including seven positive symptom, seven negative symptom, and 16 general psychopathology items (Liu et al., 2022). The total and subscale scores were calculated according to these items, with higher scores indicating greater illness severity. Cognitive function was examined using the Chinese version of the MATRICS Consensus Cognitive Battery (MCCB), encompassing 10 tests across seven domains (Liang et al., 2021).

In this study, the primary focus was on WM, which was evaluated using two individual tests covering the cognitive domains of WM: the spatial span test and the digit sequencing test, as recommended by a previous study (Wylie et al., 2019). The t-scores were computed for each test, with lower scores implying greater cognitive impairment. The PANSS and MCCB were administered twice in the study: once before the commencement of the intervention and the second instance was 3 days following the completion of the 20-session intervention.

### 2.4 Collection and processing of MRI data

MRI of the brain was conducted at the Hebei Provincial Mental Health Center using a GE SIGNA Pioneer 3.0 system (Chicago, USA) both at baseline and following a 20-session treatment regimen, with the final scan occurring on the day of the last treatment session. Patients were instructed to keep their eyes closed, avoid falling asleep during the resting-state fMRI (rs-fMRI) assessment, and minimize head movements. Structural and resting-state functional images were obtained using MPRAGE T1-weighted and gradient-echo EPI sequences, respectively. The employed scanning parameters for the T1-weighted sequence were as follows: echo time (TE) = 2.684 ms; repetition time (TR) = 6.744 ms; field of view (FOV) = 256 × 256 mm^2^; flip angle (FA) = 12°; voxel size = 1.0 × 1.0 × 1.0 mm^3^; thickness = 1.0 mm; and matrix size = 256 × 256. The echo EPI sequence parameters were as follows: TE = 30 ms; TR = 2,000 ms; FOV = 64 × 64 mm^2^; FA = 9°; axial slices = 33; matrix size = 64 × 64; slice thickness = 3.5 mm; voxel size = 3.1 × 3.1 × 3.5 mm^3^; FA = 90°; and 240 time points.

Subsequently, the acquired functional images were classified and analyzed using MRIcro software (https://www.MRIcro.com), while data preprocessing was conducted utilizing the RESTplus V1.2 toolbox (https://www.restfmri.net) on the MATLAB R2018b platform (Liu et al., 2023b). The preprocessing steps included conversion from the DICOM to NIFTI format, exclusion of the first 10 raw EPI volumes, slice timing correction, head motion correction, normalization to Montreal Neurological Institute (MNI) space using unified segmentation of the T1 images, resampling at 3 × 3 × 3 mm^3^ resolution, and smoothing with a full-width Gaussian kernel of 6 × 6 × 6 mm^3^. The six head motion parameters and the averaged signals from the cerebrospinal fluid and white matter were designated nuisance covariates and regressed out. Ultimately, all images were detrended, and the resulting images underwent further processing via bandpass filtering within the frequency range of 0.01–0.08 Hz.

### 2.5 Selection and extraction of regions of interest (ROIs)

The ROIs were selected according to previously published second-level statistical parametric mapping (SPM) analyses, which underscored the significance of frontoparietal activation in WM tasks among individuals with psychosis (Crossley et al., 2009, Deserno et al., 2012). After incorporating the Automated Anatomical Labeling (AAL) map generated by the MNI (Rolls et al., 2020), four ROIs within the frontoparietal network were included in this study. The identified ROIs were as follows: (1) the right SPL [26.11, −59.18, 62.06]; (2) the left SPL [−23.45, −59.56, 58.96]; (3) the right MFG [37.59, 33.06, 34.04]; and (4) the left MFG [−33.43, 32.73, 35.46]. Furthermore, each ROI was delineated as an 8-mm radius sphere centered on specific coordinates. An anatomical structure mask was also developed based on these regions using the AAL map to ensure precise extraction of the volume of interest (VOI). VOI extraction and subsequent spDCM analysis were implemented in SPM12 software (https://www.fil.ion.ucl.ac.uk/spm/software/spm12/). Following the standard procedure in SPM12, residuals from a general linear model (GLM) were derived from the preprocessed data. The principal eigenvariate of each VOI was extracted from the intersection of the spherical ROIs and the predefined anatomical structure mask, along with confound correction. Finally, four corresponding VOIs were generated for each patient to be used in the spDCM analysis.

### 2.6 Spectral dynamic causal modeling

The spDCM analyses were conducted using DCM12 within the SPM12 software (Friston et al., 2014). spDCM, a newly introduced DCM method for rs-fMRI, is a model-based analytical tool that estimates intrinsic causal relationships among distinct brain regions by analyzing cross-spectral density (Razi et al., 2015). The capability of spDCM to calculate endogenous coupling within regions without task-related experimental input offers enhanced computational efficiency and sensitivity to group differences, making it an appropriate approach in the current study.

According to a prior DCM study on WM (Schmidt et al., 2014), the researchers posited a consistent network architecture involving reciprocal connections between the right and left SPL and MFG. In particular, this network included intra-hemispheric connections between the SPL and MFG in both hemispheres as well as inter-hemispheric connections among the four selected ROIs Subsequently, the spDCM approach was employed to estimate the parameters of the fully connected model for each patient pre- and post-intervention. The spDCM method in this study utilized a convolution function to transform parameter estimation from the time domain of traditional DCM analysis to the frequency domain, thereby resulting in a significantly faster computational speed than that obtained in traditional DCM analysis. Following this step, a fixed-effects Bayesian model selection procedure was performed to determine the optimal model for each patient (Mao et al., 2020). Finally, a Bayesian model averaging approach was employed to evaluate the connectivity parameters, specifically focusing on the effective connection values of the optimal model (Penny et al., 2010). This model delineated the effective connectivity strength among the selected four ROIs and facilitated the subsequent phase of statistical analysis.

### 2.7 Statistical analysis

Statistical analyses were performed using IBM SPSS Statistics software version 25.0 (SPSS Inc., Chicago, IL). Continuous data, such as demographics and baseline outcome measures, were assessed via two-sample *t*-tests or Mann–Whitney U tests based on data distribution. Between-group differences in categorical data were analyzed by chi-square tests. Additionally, within-group pre-to-post-intervention changes were evaluated with paired-samples *t-*tests because the data met the normality assumptions. The effects of iTBS on clinical symptoms, neurocognitive performance, and effective connectivity within selected ROIs over a four-week period were assessed using repeated-measures analysis of variance (RMANOVA), with adjustments made for age, gender, and years of education. A factorial RMANOVA design (2 time points × 2 conditions) was employed to identify significant interactions between time (baseline and 4 weeks) and group (iTBS and sham-iTBS). A p-value < 0.05 (two-sided) was considered to indicate statistical significance. Following the methodology outlined by a previous study (Wobrock et al., 2015), effect sizes were computed based on the changes in scores from the baseline for each group, utilizing an online tool (Lenhard and Lenhard, 2016). Cohen's *d* values were used to interpret the effect sizes, with thresholds defined as small (0.2–0.5), medium (0.5–0.8), and large (≥0.8) (Hou et al., 2024). Furthermore, a stepwise linear regression analysis was performed to examine the relationship between significant effective connectivity and clinical measures in the active group. The alterations in effective connectivity pre- and post-intervention were considered as the dependent variables, while the variations in clinical and cognitive test parameters before and after the iTBS intervention were treated as the independent variables. Finally, the *p*-values derived from the correlation analysis were adjusted using the Bonferroni correction method.

## 3 Results

### 3.1 Demographics and baseline characteristics

**Fig. 1:**
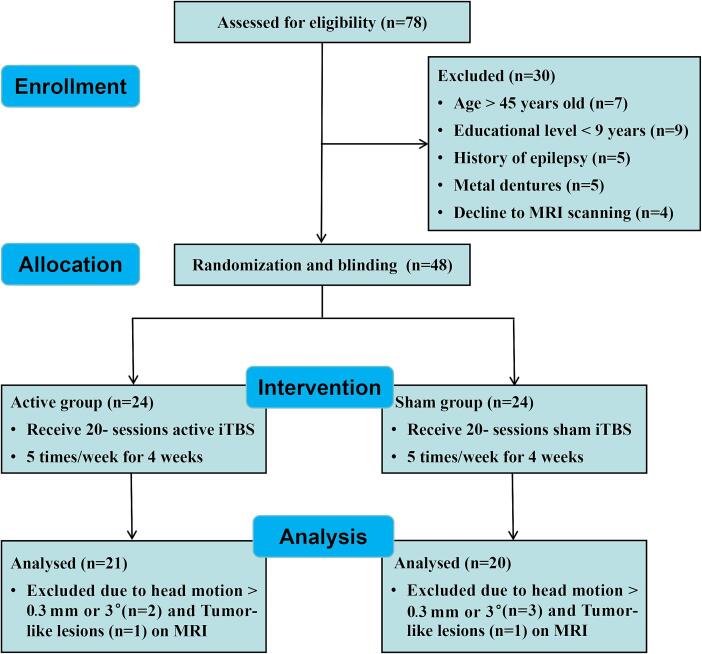
The selection process of the participants.

Between May 2023 and January 2024, 78 potential patients underwent screening to determine their study eligibility. Of these patients, 30 were excluded due to factors including advanced age (*n* = 7), low educational level (*n* = 9), epilepsy history (*n* = 5), the presence of metal dental implants (*n* = 5), and refusal to undergo MRI scanning (*n* = 4). Subsequently, the remaining 48 patients who met the eligibility criteria were randomly allocated to either the active (*n* = 24) or sham (*n* = 24) iTBS groups. Two patients, one from each group, were excluded from the analysis due to the presence of tumor-like lesions. Additionally, five patients, comprising two from the active group and three from the sham group, were excluded owing to head movements exceeding a mean frame-to-frame displacement of 0.3 mm,

A flowchart illustrating the patient exclusion process is presented in Fig. 1**,** while details on sample size calculation are provided in Supplementary text 2. Furthermore, the medication information, RMT and stimulus intensity of the 41 patients included in the statistical analysis are displayed in **Supplementary** Table S1**.**

As demonstrated in Table 1, the active and sham groups did not exhibit significant differences in demographic and baseline characteristics, including age, gender, education years, age at first onset, MCCB total score, MCCB WM score, PANSS positive score, PANSS negative score, and PANSS general psychopathology score (all *p* > 0.05).

**Table 1:** Demographics and baseline characteristics.

Characteristics	Active (n = 21)	Sham (n = 20)	*t/X^2^*	*p*
Age (years)	30.29 ± 7.91	32.15 ± 6.23	−0.836	0.408
Gender (M/F)	10/11	9/11	0.028	0.867
Education (years)	13.52 ± 2.77	12.25 ± 2.40	1.570	0.124
Age at first onset	21.76 ± 6.09	24.85 ± 6.38	−1.585	0.121
PANSS_positive	12.29 ± 2.45	13.50 ± 3.36	−1.325	0.193
PANSS_negative	17.81 ± 3.16	17.00 ± 4.04	0.717	0.418
PANSS_GP	31.57 ± 3.93	29.45 ± 4.88	1.536	0.133
MCCB total score	35.57 ± 8.68	33.80 ± 9.67	0.618	0.540
MCCB_WM	40.00 ± 7.62	41.75 ± 9.32	−0.660	0.513

**Abbreviation**

M: male; F: female; PANSS: Positive and Negative Symptom Scale; MCCB, MATRICS Consensus Cognitive Battery; WM, working memory; GP, general psychopathology.

### 3.2 Effects of iTBS on clinical symptoms and WM deficits

**Fig. 2:**
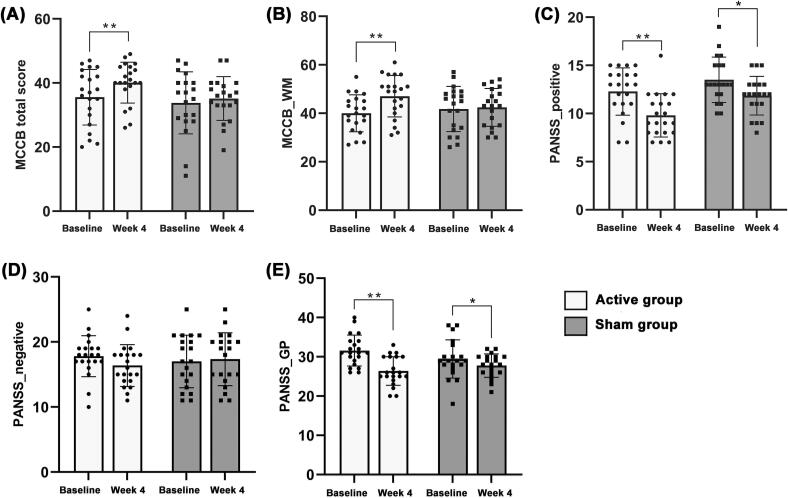
Clinical and neuropsychological performances after 4 weeks of treatment between the iTBS and sham-iTBS groups. (A) MCCB total score. (B) MCCB working memory. (C) PANSS_positive. (D) PANSS_negative. (E) PANSS general psychopathology.

After four weeks of treatment, a two-way RMANOVA showed significant time × group effects (interaction) in the MCCB total (*F* = 3.927, *p* = 0.045, Cohen’s *d* = 0.340), MCCB_WM (*F* = 12.784, *p* = 0.001, Cohen’s *d* = 0.733), PANSS_negative (*F* = 4.904, *p* = 0.033, Cohen’s *d* = 0.483), and PANSS general psychopathology scores (*F* = 6.530, *p* = 0.015, Cohen’s *d* = 0.775) (Table 2 and Fig. 2). Additionally, the active group demonstrated significant pre-to-post-intervention differences in the MCCB total, MCCB_WM, PANSS_positive, and PANSS general psychopathology scores (all *p* < 0.01); in the case of the sham group, significant differences were found in the PANSS positive and PANSS general psychopathology scores before and after the intervention (all *p* < 0.05) (Table 2 and Fig. 2).

**Table 2:** Clinical and neuropsychological performances after 4 weeks of treatment between the iTBS and sham-iTBS groups.

**Variable Names**	**Active**		**Sham**		**Results of RMANOVA**		**Within-group comparison**	
	**Pre**	**Post**	**Pre**	**Post**	**Time-by-group Interaction (*F, p*)**	**Effect sizes** **(Cohen's *d*)**	**Active (*t, p*)**	**Sham (*t, p*)**
MCCB total score	35.57 ± 8.68	40.10 ± 6.37	33.80 ± 9.67	35.15 ± 6.81	(3.927, 0.045)	0.340	(−3.934, 0.001)	(−1.149, 0.265)
MCCB_WM	40.00 ± 7.62	47.05 ± 8.56	41.75 ± 9.32	42.45 ± 7.80	(12.784, 0.001)	0.733	(−6.641, < 0.001)	(−0.487, 0.632)
PANSS_positive	12.29 ± 2.45	9.81 ± 2.25	13.50 ± 3.36	11.85 ± 2.01	(1.092, 0.303)	0.278	(5.630, < 0.001)	(2.477, 0.023)
PANSS_negative	17.81 ± 3.16	16.38 ± 3.32	17.00 ± 4.04	17.35 ± 4.06	(4.904, 0.033)	0.483	(1.878, 0.075)	(−1.789, 0.090)
PANSS_GP	31.57 ± 3.93	26.38 ± 3.65	29.45 ± 4.88	27.75 ± 2.99	(6.530, 0.015)	0.775	(5.171, < 0.001)	(1.846, 0.041)

**Abbreviation**

RMANOVA, repeated-measures analysis of variance.

### 3.3 Adverse effects

Two patients in the active group reported experiencing mild dizziness and nausea following the first treatment, which subsided with rest and allowed for the completion of subsequent treatments.

### 3.4 Bayesian model selection results

**Fig. 3:**
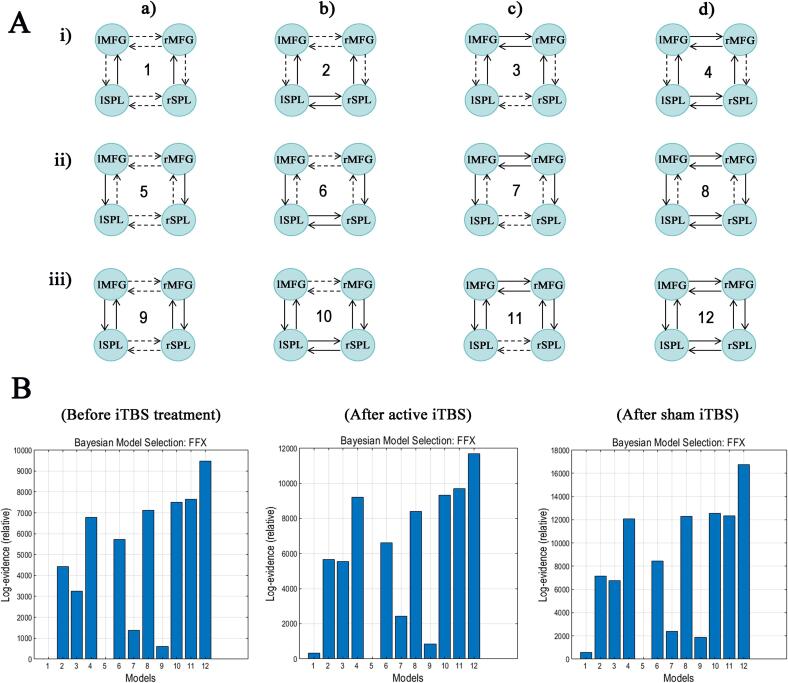
(A) Model space tested in this study. Abbreviations: SPL: superior parietal lobule; MFG: right middle frontal gyrus; FFX: fixed effects. (B) Bayesian model selection (BMS) among all 12 dynamic causal models (DCMs) over all groups.

Based on prior research findings (Schmidt et al., 2013, Schmidt et al., 2014), we hypothesized the presence of four potential hemispheric connections: no connections, interhemispheric connections between parietal areas, interhemispheric connections between frontal areas, and both (Fig. 3**A, a-d**). The parieto-frontal connections are observed in the first row (Fig. 3**A, i)**, frontoparietal connections in the second row (Fig. 3**A, ii**), and bidirectional connections in Fig. 3**A, iii**. Consequently, our model space comprised 12 alternative models, with each fitted to the data of individual patients. At the group level, the fully connected model (model 12) was identified as the optimal model across all groups (Fig. 3**B**).

### 3.5 Between-group differences in effective connectivity

**Fig. 4:**
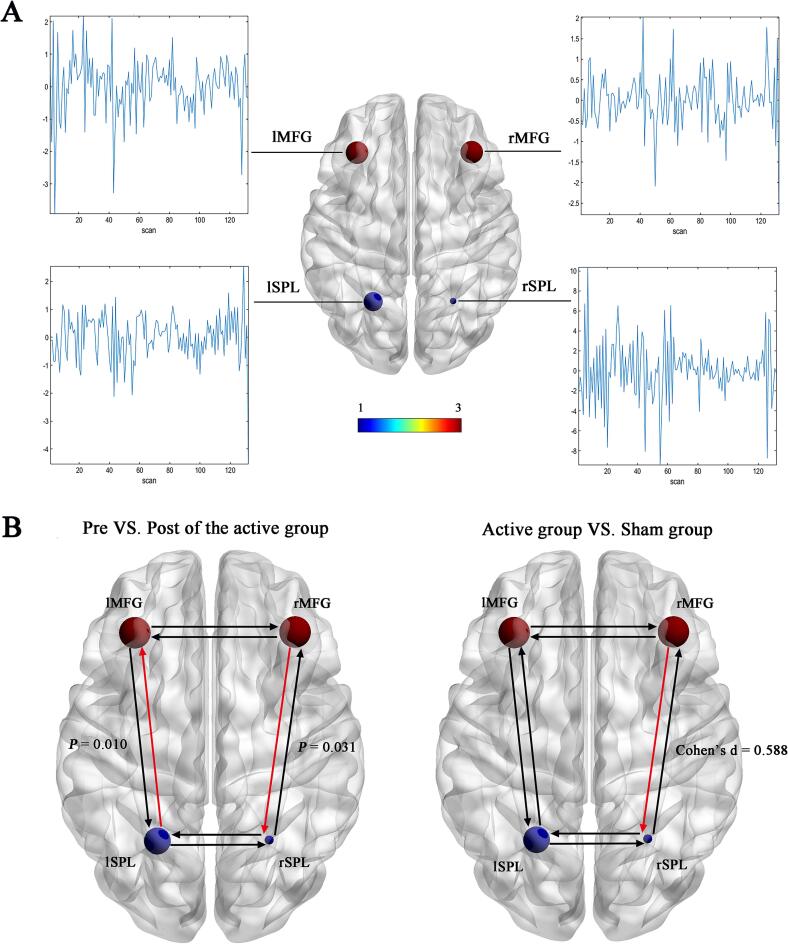
(A) Time series of regions of interest (ROIs); (B) Effective connectivity difference between groups. The red line indicated increased connectivity.

The time series data from the four selected ROIs employed for spDCM inversion are presented in Fig. 4**A**. Following the iTBS intervention, in terms of the effect size, only the effective connectivity of right MFG to right SPL reached a medium level (Cohen’s *d* = 0.588), with a significant time × group effect (interaction, *F* = 4.857, *p* = 0.017), while the rest were less than 0.2. Furthermore, the active group demonstrated a significant increase in connectivity strengths from the right MFG to the right SPL and from the left SPL to the left MFG compared to the pre-treatment levels (all *p* < 0.05). As for the sham group, no significant difference was found in the fully connected model before and after the intervention (Fig. 4**B** and **Supplementary** Table S2).

### 3.6 Correlations between effective connectivity and symptoms

Considering that the active group exhibited improved cognitive test scores (MCCB total and MCCB_WM scores) post-intervention, we conducted a stepwise linear regression analysis to investigate the potential associations between alterations in effective connectivity and changes in various clinical and cognitive test parameters before and after the iTBS intervention. However, our results found no significant relationships between these variabless (Table 3).

**Table 3:** Regression analysis between the effective connectivity estimate and demographic and clinical parameters.

Items	*t, p* (rMFG to rSPL connectivity)	*t, p* (lSPL to lMFG connectivity)
MCCB total score	0.164, 0.872	−1.119, 0.281
MCCB_working memory	−0.687, 0.503	−0.655, 0.522
PANSS_positive	−0.462, 0.651	−1.315, 0.208
PANSS_negative	−0.344, 0.736	−0.826, 0.422
PANSS_general psychopathology	0.616, 0.547	0.352, 0.730

Note: No significant relationship between effective connectivity estimate and relevant demographical and clinical variables was found. The first column in the table was the *t* value, and the second column next to it was the corresponding *p*-value. *P-*value less than 0.05 was considered statistically significant (uncorrected).

## 4 Discussion

To our knowledge, this study is the first randomized controlled trial (RCT) to investigate the clinical and neuropsychological outcomes of parietal iTBS in patients with SZ as well as examine its effects on resting-state effective connectivity within the frontoparietal network using the spDCM approach. Our study results revealed that active iTBS led to significant enhancements in WM, positive symptoms, negative symptoms,and general psychopathology, along with effect sizes from small to medium in patients presenting with stable psychiatric symptoms. Moreover, parietal iTBS significantly augmented the connectivity strengths from the right MFG to the right SPL and from the left SPL to the left MFG. All these findings support the clinical efficacy of parietal iTBS for ameliorating WM deficits and highlight its modulatory effects on the effective connectivity in the frontoparietal network.

Currently, limited effective interventions are available for managing visual-spatial WM deficits in individuals with SZ. A previous investigation evaluated the application of neuronavigation technology to target the left DLPFC in patients with SZ who were randomly assigned to receive either iTBS or sham treatment over 2 weeks (Wang et al., 2022). The study findings showed that the iTBS group exhibited notable enhancements in 3-back accuracy when compared to the test results of the sham group. Furthermore, iTBS was demonstrated to modulate the activity of the right occipital areas and right precuneus. However, comparing the results of other n-back tests between the two groups before and after intervention did not reveal any significant differences. Research on healthy populations has shown that the functioning of the parietal and prefrontal areas is essential for information storage and is closely linked to behavioral responses. Additionally, rTMS treatment was found to strengthen the connectivity between these regions (Li et al., 2017). Other studies have postulated that dysfunctional parietal lobe activity may be the underlying factor for visual-spatial WM deficits in individuals with SZ (Benson and Park, 2013). In this study, we utilized the left P3 region as the stimulation target and demonstrated the therapeutic efficacy of iTBS in alleviating WM impairments in patients with SZ, along with favorable safety profiles.

Rs-fMRI can assess brain functional connectivity in individuals without task performance, making it particularly valuable in patients with cognitive impairment or those unable to engage in task-based assessments (Abdul Wahab et al., 2022). Compared to task-based fMRI, rs-fMRI offers several advantages, including enhanced repeatability, controllability, and reduced interference from extraneous factors (Bennett and Miller, 2013). Our study identified predominant connectivity patterns between the parietal and frontal lobes in the active and sham groups, consistent with the findings from previous task-based fMRI research (Schmidt et al., 2014). A prior investigation on the general population revealed that functional connectivity between the regions associated with conventional WM, such as the mid-frontal and parietal cortices, was heightened during n-back tasks (Liang et al., 2013). In addition, bidirectional transport has been noted between the frontal and parietal regions across varied populations, including the general population, high-risk individuals, and patients with SZ (Schmidt et al., 2014, Schmidt et al., 2013).

DCM has been established as a viable method for examining synaptic plasticity, with research suggesting that disrupted brain connectivity in SZ may stem from dysregulated N-methyl-D-aspartate receptor-dependent synaptic plasticity, a phenomenon influenced by neuromodulatory transmitters such as dopamine (Stephan et al., 2009). Moreover, previous investigations employing cognitive tasks, specifically those utilizing the n-back task in conjunction with DCM, have demonstrated a progressive reduction in the modulation of the connectivity between the mid-frontal and upper parietal lobes of the right hemisphere in healthy individuals, those at risk for mental illness, and those experiencing their first psychiatric episode (Schmidt et al., 2013, Schmidt et al., 2014). This observed reduction in connectivity may be attributed to the previously discussed compromised synaptic plasticity within the frontal and parietal brain regions.

Our study revealed that patients in the active group exhibited a significant enhancement in connectivity strengths from the right MFG to the right SPL after 20 sessions of iTBS treatment compared with the sham group. Researchers have proposed that connections originating from the parietal cortex to the frontal cortex may be involved in encoding incoming stimuli, whereas connections from the frontal cortex to the parietal cortex are likely to facilitate the updating of rules (Gazzaley et al., 2004, Sauseng et al., 2005). A previous fMRI study showed that rule updates preferentially activate the prefrontal cortex, while stimulus updates activate the parietal cortex of healthy participants (Montojo and Courtney, 2008). Furthermore, a previous study identified the right MFG as a crucial role in facilitating functional integration within the WM network in patients with schizophrenia (Schmidt et al., 2013). In our study, we selected the P3 region of the parietal lobe as the site for stimulation. The observed enhancement in effective connectivity from the right MFG to the right SPL may indirectly corroborate the key role of the right MFG in the WM network. Notably, we also observed that, within the active group, the connection strength from the left SPL to the left MFG was augmented relative to baseline measurements. Research integrating TMS with fMRI has demonstrated that TMS can induce measurable effects at both local and remote cortical sites relative to the stimulation location (Wang et al., 2014). This finding implies that the effects of TMS should be understood within the framework of a more interconnected global neural network. For instance, Davis et al. observed that the application of 1 Hz rTMS facilitated increased global connectivity across various brain modules bilaterally distant from the stimulation site in older adults (Davis et al., 2017). Within a Bayesian inference framework, prominent models of schizophrenia suggest that patients disproportionately prioritize incoming sensory evidence over prior beliefs, leading to an increased perception of novelty and a persistent state of surprise (Adams et al., 2013). If the connections from the parietal cortex to the frontal cortex are implicated in the encoding of incoming stimuli, the enhancement of effective connectivity from the right MFG to the right SPL may be associated with atypical novelty processing in individuals with psychiatric disorders. However, further research is necessary to substantiate this hypothesis. A recent literature review proposed that rTMS could modulate excitability and plasticity within a specific cortical area, thereby eliciting broader effects on interconnected networks at a systemic level (Jannati et al., 2023). Additionally, the proposed mechanism underlying the therapeutic benefits of rTMS is based on dysfunction regulation within and among functional networks (Cash et al., 2021a; Siddiqi et al., 2020). Consequently, rTMS targeting of structural and/or functional networks has emerged as a critical area of interest for enhancing rTMS effectiveness in therapeutic applications.

It was worth noting that in the sham group, significant differences were found in the PANSS positive and PANSS general psychopathology scores before and after the intervention. In present, sham TMS approaches are widely used in basic and clinical research to ensure that observed effects are due to the intended neural manipulation instead of being caused by various possible side effects (Duecker and Sack, 2015). However, as TMS therapy for mental illness is gradually recognized by the public, this may lead to a sustained increase in the placebo effect of sham TMS. In the future, a comprehensive understanding of the physiological and psychological mechanisms underlying the placebo effect of sham TMS could significantly enhance the development of more effective treatment strategies for schizophrenia. Furthermore, we did not detect any potential associations between alterations in connectivity strength and the various clinical and cognitive test parameters before and after iTBS intervention in the active group. This may be related to the relatively smaller sample size and shorter intervention course of this study.

Our study has several limitations that should be considered. First, the small sample size due to resource constraints may restrict the generalizability of our findings; therefore, these study results should be cautiously interpreted. Second, we did not explore the long-term effects of parietal iTBS on the resting-state effective connectivity in the frontoparietal network. Hence, subsequent research should incorporate extended follow-up evaluations to address this gap. Third, owing to the long duration required for the PANSS test and MCCB test, we did not allocate additional time to assess the general mental health effects of iTBS on patients with schizophrenia, including factors such as anxiety and depression. Last, although additional brain regions, such as the anterior cingulate cortex, are known to be involved in WM processes (Ma et al., 2012), the present study model did not include these regions to ensure parsimony and comparability with previous studies of patients with SZ (Schmidt et al., 2013, Schmidt et al., 2014).

In conclusion, this study revealed that 20 sessions of parietal iTBS over 4 weeks might significantly improve clinical symptoms and WM deficits in patients with SZ. The iTBS intervention also induced changes in resting-state effective connectivity within the frontoparietal network among these patients. Our study findings demonstrate that parietal iTBS may serve as a promising treatment strategy for alleviating cognitive impairment in patients with SZ.

## CRediT authorship contribution statement

**Li Li:** Writing – original draft, Software, Formal analysis. **Lina Wang:** Writing – review & editing, Methodology. **Han Wu:** Writing – review & editing, Methodology. **Bing Li:** Writing – review & editing, Data curation. **Weigang Pan:** Writing – review & editing, Formal analysis. **Wenqing Jin:** Writing – review & editing, Data curation. **Wen Wang:** Writing – review & editing. **Yanping Ren:** Writing – review & editing, Supervision. **Chaomeng Liu:** Validation, Supervision, Resources, Methodology, Investigation, Data curation, Conceptualization. **Xin Ma:** Validation, Supervision, Resources, Methodology, Investigation, Data curation, Conceptualization.

## Declaration of Competing Interest

The authors declare that they have no known competing financial interests or personal relationships that could have appeared to influence the work reported in this paper.

## Data Availability

Data will be made available on request.
