# Supplementary material for: Effects of parietal iTBS on resting-state effective connectivity within the frontoparietal network in patients with schizophrenia: An fMRI study
Source: Neuroimage Clin. 2024 Nov 26;45:103715. doi: 10.1016/j.nicl.2024.103715 (PMC11638604; doi:10.1016/j.nicl.2024.103715)
Supplement: Supplementary Data 1 [file mmc1.doc]

***Supplementary Materials***

# Effects of parietal intermittent theta burst stimulation on resting-state effective connectivity within the frontoparietal network in patients with schizophrenia: An fMRI study

# **Supplementary Text 1.** Positioning methods for P3

The 10-20 system electrode placement method is the standard electrode placement method specified by the International Electroencephalography Society. Initially, two baselines are established on the surface of the scalp: one being the anterior-posterior line extending from the nasal root to the occipital protuberance, constituting 100% of the total length; the other being the line connecting the anterior fovea of both ears, also accounting for 100% of the total length. The point of intersection between these two lines at the apex of the head designates the location of the Cz electrode. The distance from the midpoint of the forehead to the nasal root and the distance from the occipital point to the posterior occipital protuberance each represent 10% of the total length of this line, with the remaining points spaced at intervals of 20% of the total length.

The participants were seated in a comfortable chair while wearing commercial standard extended 10-20 EEG stretch caps for 39 channels, which were designed in accordance with the 10-20 system to define the positions. These EEG caps took into consideration the relative distances of 10% or 20% of the individual distances from the vertex to the relevant landmarks, requiring precise adaptation of the cap to these landmarks due to its stretching nature. The cap sizes were chosen based on the unique head dimensions of each individual and were then applied to the participants' heads by a skilled EEG technician and a helper. The stimulation of P3 primarily affected Brodmann areas 40 and to a lesser extent, Brodmann area 7, within the inferior and posterior parietal lobe near the intraparietal sulcus.

**Reference**

Herwig, U., Satrapi, P., Schönfeldt-Lecuona, C., 2003. Using the international 10-20 EEG system for positioning of transcranial magnetic stimulation. Brain Topogr 16, 95-99.

# **Supplementary Text 2.** Sample size calculation

To calculate sample size, we referred to the literature that investigated the effect of iTBS on working memory deficits in patients with schizophrenia (wang et al., 2022). In this study, they reported an effect size of about 0.9 in the 3-back test following a 14 consecutive days of active rTMS targeting the left dorsolateral prefrontal cortex. Therefore, we used the effect size of 0.9 to calculate the sample size with a significance level of 0.05 and 80% detection power using Gpower Version 3.1.9.2, resulting in a sample size of 42 (21 subjects for each group). Given the high compliance of patients with stable symptoms, we expected a dropout rate of 10%-15% and set the total number of required participants for this trial between 47 and 50.

**Reference**

Wang, L., Li, Q., Wu, Y., Ji, G.J., Wu, X., Xiao, G., Qiu, B., Hu, P., Chen, X., He, K., Wang, K., 2022. Intermittent theta burst stimulation improved visual-spatial working memory in treatment-resistant schizophrenia: A pilot study. J Psychiatr Res 149, 44-53.

***Supplementary Table 1. Medication information and RMT of the 41 patients included in the statistical analysis.***

| **Patients** | **Psychiatric drugs** | **RMT** | **Power (%)** |
| --- | --- | --- | --- |
| Patient 1 | Risperidone 6mg/day | 56 | 45 |
| Patient 2 | Olanzapine 20mg/day | 52 | 42 |
| Patient 3 | Olanzapine 15mg/day | 54 | 43 |
| Patient 4 | Clozapine 200 mg/day, Amsulpride 0.8g/day | 52 | 42 |
| Patient 5 | Clozapine 300mg/day, Amsulpride 0.6g/day | 62 | 50 |
| Patient 6 | Olanzapine 15mg/day, Paliperidone 3mg/day | 56 | 45 |
| Patient 7 | Amsulpride 0.6g/day | 56 | 45 |
| Patient 8 | Risperidone 4mg/day, Clozapine 400mg/day | 52 | 42 |
| Patient 9 | Aripiprazole 10mg/day | 58 | 46 |
| Patient 10 | Aripiprazole 20mg/day | 58 | 46 |
| Patient 11 | Bunanserin 16mg/day, Quetiapine 400mg/day | 60 | 48 |
| Patient 12 | Risperidone 4mg/day | 62 | 50 |
| Patient 13 | Clozapine 200mg/day | 60 | 48 |
| Patient 14 | Risperidone 4mg/day | 60 | 48 |
| Patient 15 | Olanzapine 5mg/day, Pirobirone 48mg/day | 58 | 46 |
| Patient 16 | Clozapine 300mg/day | 60 | 48 |
| Patient 17 | Clozapine 300mg/day, Aripiprazole 20mg/day | 60 | 48 |
| Patient 18 | Bunanserin 20mg/day | 56 | 45 |
| Patient 19 | Risperidone 3mg/day, Clozapine 225mg/day | 56 | 45 |
| Patient 20 | Paliperidone 9mg/day | 62 | 50 |
| Patient 21 | Risperidone 5.5mg/day, Aripiprazole 25mg/day | 62 | 50 |
| Patient 22 | Olanzapine 10mg/day, Lurasidone 80mg/day | 52 | 42 |
| Patient 23 | Bunanserin 20mg/day | 56 | 45 |
| Patient 24 | Risperidone 3mg/day, Bunanserin 24mg/day | 56 | 45 |
| Patient 25 | Risperidone 3mg/day | 60 | 48 |
| Patient 26 | Ziprasidone 40mg/day, Clozapine 50mg/day | 62 | 50 |
| Patient 27 | Risperidone 6mg/day | 58 | 46 |
| Patient 28 | Risperidone 4mg/day | 52 | 42 |
| Patient 29 | Clozapine 200mg/day | 56 | 45 |
| Patient 30 | Clozapine 15mg/day, Risperidone 6mg/day | 56 | 45 |
| Patient 31 | Aripiprazole 20mg/day | 52 | 42 |
| Patient 32 | Clozapine 150mg/day, Bunanserin 16mg/day | 58 | 46 |
| Patient 33 | Risperidone 4mg/day | 58 | 46 |
| Patient 34 | Olanzapine 15mg/day | 62 | 50 |
| Patient 35 | Bunanserin 16mg/day, Aripiprazole 20mg/day | 58 | 46 |
| Patient 36 | Aripiprazole 25mg/day, Clozapine 75mg/day | 56 | 45 |
| Patient 37 | Clozapine 200mg/day | 52 | 42 |
| Patient 38 | Risperidone 5mg/day | 56 | 45 |
| Patient 39 | Olanzapine 15mg/day | 62 | 50 |
| Patient 40 | Bunanserin 8mg/day, Aripiprazole 5mg/day | 58 | 46 |
| Patient 41 | Olanzapine 15mg/day | 60 | 48 |

Note: RMT, resting motor threshold; Power, stimulus intensity. Patients 1-21 were in the active group, while patients 22-41 belonged to the sham group. In accordance with the 2-second on and 8-second off cycle, a total of 600 pulses (approximately 190 seconds in duration) will be administered at 80% of the RMT in the active group. For the Super Rapid 2 protocol, the maximum transcranial magnetic stimulation (TMS) intensity can reach up to 50% of the maximum output with a 50Hz stimulation frequency. In instances where a participant's RMT exceeds 62%, the maximum stimulus intensity will be capped at 50%.

***Supplementary Table 2. Effective connectivity parameters differences after 4 weeks of treatment between the iTBS and sham-iTBS groups.***

| **Variable Names** | **Active** | | **Sham** | | **Results of RMANOVA** | | **Within-group comparison** | |
| --- | --- | --- | --- | --- | --- | --- | --- | --- |
|  | **Pre** | **Post** | **Pre** | **Post** | **Time-by-group Interaction (*F, p*)** | **Effect sizes**  **(Cohen's *d*)** | **Active (*t, p*)** | **Sham (*t, p*)** |
| lSPL to rSPL | 0.384 ± 0.325 | 0.403 ± 0.426 | 0.468 ± 0.376 | 0.475 ± 0.347 | (0.025, 0.875) | 0.034 | (-0.245, 0.783 ) | (-0.092, 0.813) |
| rMFG to rSPL | 0.074 ± 0.360 | 0.250 ± 0.218 | 0.035 ± 0.268 | 0.020 ± 0.357 | (4.857, 0.017) | 0.588 | (-2.332, 0.031) | (0.184, 0.840) |
| rSPL to lSPL | 0.068 ± 0.424 | 0.107 ± 0.487 | 0.046 ± 0.413 | 0.058 ± 0.346 | (0.074, 0.416) | 0.063 | (-0.346, 0.804) | (-0.102, 0.846) |
| lMFG to lSPL | 0.278 ± 0.346 | 0.372 ± 0.389 | 0.306 ± 0.427 | 0.346 ± 0.395 | (1.104, 0.125) | 0.137 | (-0.892, 0.314) | (-0.415, 0.626) |
| rSPL to rMFG | 0.624 ± 0.572 | 0.725 ± 0.545 | 0.542 ± 0.453 | 0.602 ±0.426 | (1.332, 0.104) | 0.078 | (-0.624, 0.456) | (-0.447, 0.527) |
| lMFG to rMFG | 0.247 ± 0.432 | 0.317 ± 0.456 | 0.358 ± 0.478 | 0.404 ± 0.572 | (1.187, 0.121) | 0.052 | (-0.617, 0.504) | (-0.378, 0.632) |
| lSPL to lMFG | 0.068 ± 0.283 | 0.292 ± 0.155 | 0.094 ± 0.247 | 0.268 ± 0.310 | (2.346, 0.067) | 0.184 | (-2.837, 0.010) | (-2.015, 0.052) |
| rMFG to lMFG | 0.072 ± 0.245 | 0.077 ± 0.276 | 0.087 ± 0.284 | 0.095 ± 0.312 | (0.021, 0.896) | 0.026 | (-0.073, 0.915) | (-0.089, 0.904) |

Note: *P-*value less than 0.05 was considered statistically significant (uncorrected).
